# Phosphaturic mesenchymal tumor in the temporalis muscle causing tumor-induced osteomalacia: a case report and literature review

**DOI:** 10.3389/fmed.2026.1807111

**Published:** 2026-05-14

**Authors:** Bo Gao, Chao Wang, Xiaoming Xu, Xiuxiu Lai, Xiaohong Xu

**Affiliations:** 1 Department of Prosthodontics, Second Affiliated Hospital of Zhejiang University School of Medicine, Hangzhou, Zhejiang, China; 2 Stomatology Hospital, School of Stomatology, Zhejiang University School of Medicine, Zhejiang Provincial Clinical Research Center for Oral Diseases, Key Laboratory of Oral Biomedical Research of Zhejiang Province, Cancer Center of Zhejiang University, Engineering Research Center of Oral Biomaterials and Devices of Zhejiang Province, Hangzhou, Zhejiang, China; 3 Department of Radiology, Second Affiliated Hospital, Zhejiang University School of Medicine, Hangzhou, Zhejiang, China; 4 Department of Pathology, Second Affiliated Hospital, Zhejiang University School of Medicine, Hangzhou, Zhejiang, China; 5 Geriatric Medicine Center, Department of Geriatric Medicine, Zhejiang Provincial People’s Hospital, Affiliated People’s Hospital, Hangzhou Medical College, Hangzhou, Zhejiang, China; 6 Department of Cardiology, Zhejiang Hospital, Hangzhou, Zhejiang, China

**Keywords:** osteomalacia, PET/CT, phosphaturic mesenchymal tumor, temporalis muscle, tumor-induced osteomalacia

## Abstract

Tumor-induced osteomalacia (TIO) is a rare paraneoplastic syndrome characterized by renal phosphate wasting, hypophosphatemia, and impaired bone mineralization, primarily caused by phosphaturic mesenchymal tumors (PMTs). Due to the small size and non-specific clinical presentation, PMTs are often difficult to localize, resulting in delayed diagnosis. This study presents a case of a 54-year-old man who experienced a 2-year history of progressive polyarthralgia affecting the knees, hips, and lower back, which exacerbated over the past 3 months. Laboratory tests revealed hypophosphatemia, elevated alkaline phosphatase (ALP) levels, and increased parathyroid hormone (PTH) levels. Moreover, a dual-energy X-ray absorptiometry (DXA) scan indicated low bone mineral density (BMD), while a whole-body bone scintigraphy revealed multiple rib pathological fractures, indicative of osteomalacia. Although serum fibroblast growth factor 23 (FGF23) was not measured, the clinical and biochemical profiles were highly consistent with tumor-induced osteomalacia (TIO). Subsequent Gallium-68 1,4,7,10-tetraazacyclododecane 1,4,7,10-tetraacetic acid tyrosine-3-octreotate positron emission tomography/computed tomography (^68^Ga-DOTA-TATE) positron emission tomography/computed tomography (PET/CT) confirmed a hypermetabolic lesion in the left temporalis muscle. The lesion was completely resected, and histopathological examination confirmed the diagnosis of PMT, with positive immunostaining for special AT-rich sequence-binding protein 2 (SATB2), neural cell adhesion molecule (CD56), and somatostatin receptor type 2 (SSTR2). No tumor recurrence was observed during follow-up at 6 months after surgery. Clinicians should consider occult PMTs in patients with unexplained hypophosphatemia, bone pain, or pathological fractures, and employ functional imaging modalities such as ^68^Ga-DOTA-TATE PET/CT for early tumor localization and potential cure through resection.

## Introduction

Tumor-induced osteomalacia (TIO) is a rare paraneoplastic syndrome commonly caused by phosphaturic mesenchymal tumors (PMTs) (1, 2). The annual incidence of TIO has been estimated to be 0.13 per 100,000 individuals according to an epidemiological study from Denmark (3). PMTs are rare, typically benign tumors that secrete excessive fibroblast growth factor-23 (FGF-23). This leads to renal phosphate wasting, hypophosphatemia, and impaired bone mineralization (1, 2). The clinical presentation is non-specific and my include bone pain, muscle weakness, and pathological fractures (4). PMTs pose a significant diagnostic challenge, resulting in a substantial diagnostic delay that averages 4.8 ± 4.61 years (5).

These tumors can arise in both soft and osseous tissues, with a predilection for the extremities and the head and neck region (6, 7). However, tumor localization is challenging because of the small size and potential to occur anywhere in the body. Conventional anatomical imaging modalities often exhibit limited diagnostic accuracy and typically rely on the guidance from the functional imaging techniques. Among various techniques, positron emission tomography/computed tomography (PET/CT) using ^68^Ga-DOTA-TATE has demonstrated superior sensitivity and specificity in detecting PMTs (8). In a study involving 56 patients with suspected TIO, this technique demonstrated 95.13% sensitivity and 60.00% specificity (9).

However, no cases of PMT occurring in the temporalis muscle have been reported in the literature. This study presents a case with PMT in the left temporalis muscle that manifested as TIO, detected using ^68^Ga-DOTA-TATE PET/CT, thereby expanding the documented anatomical spectrum of this rare neoplasm.

## Case presentation

A 54-year-old man was referred to the orthopedics department with unexplained polyarticular pain that had persisted for 2 years. The polyarthralgia affected his knees, hips, and lower back. During this period, the patient underwent lumbar spine surgery, followed by unexplained impaired bone healing. Meanwhile, the patient required a mobility aid for walking due to osteonecrosis in the left femoral head. His lower back and buttock pain intensified in the last 3 months, which included nocturnal pain and difficulty turning over in bed.

Physical examination revealed local tenderness in the knee joint and limited range of motion in the right knee and hip joints. Biochemical results revealed hypophosphatemia with serum phosphate levels of 0.29 mmol/L (0.85–1.51), low 25-hydroxy vitamin (25 (OH)D) level of 18.9 μg/l (20–50), increased alkaline phosphatase (ALP) level of 214 U/L (45–125), and increased parathyroid hormone (PTH) level of 150 pg./mL (15–65). The preoperative 24-h urinary phosphate level was 15.75 mmol/24 h. Based on the relevant tests, the preoperative tubular reabsorption of phosphate (TRP) and tubular maximum reabsorption of phosphate to glomerular filtration rates (TmP/GFR) were 58.07% and 0.17 mmol/L, respectively. A complete blood count, along with tests for hepatic and renal function, showed results within normal ranges. Dual-energy X-ray absorptiometry (DXA) indicated low bone mineral density, which was diagnosed as osteoporosis. Whole-body bone scintigraphy using technetium-99 m methylene diphosphonate (99mTc-MDP) also revealed multiple foci of intensely increased osteoblastic activity in the bilateral multiple ribs and costochondral junctions, demonstrating fractures consistent with osteomalacia and associated pathological fractures (Figures 1A,B).

**Figure 1:**
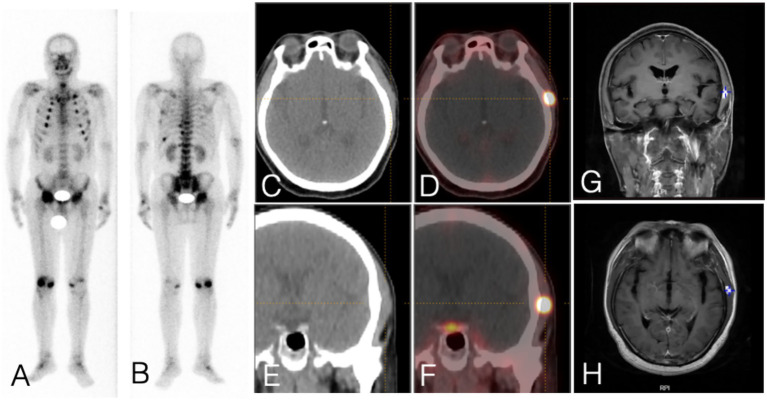
Whole-body bone scintigraphy with ^99m^Tc-MDP scan showing a dense shadow of radioactivity distribution in bilateral ribs, costochondral junctions, right shoulder, L4/L5 vertebrae, bilateral femoral heads, bilateral knees, and bones of the feet **(A,B)**. PET/CT whole-body octreotide imaging: after intravenous administration of ^68^Ga-DOTA-TATE and 60 min resting, whole-body PET/CT tomography was performed. A soft-tissue mass measuring approximately 1.5 cm × 1.1 cm with abnormally high radioactivity was detected in the left temporalis muscle, indicating an SUV max of 66.3 **(C–F)**. Enhanced MRI demonstrated a 7 mm *10 mm mass in left temporalis muscle **(G,H)**.

The patient was hospitalized for hypophosphatemic osteomalacia and received treatment with calcitriol and neutral phosphorus solution for symptomatic management. After 7 days of treatment, the patient’s bone pain had resolved. Laboratory tests revealed that hypophosphatemia improved (0.61 mmol/L), while the ALP and PTH levels decreased to 194 U/L and 83.2 pg./mL, respectively (Table 1).

**Table 1:** Biochemical test results before and after tumor resection.

Parameters	Initial admission	Before surgery	3 days after surgery	27 days after surgery
Serum K (3.5–5.3 mmol/L)	–	3.51	4.33	4.13
Serum Na (137–147 mmol/L)	–	141.4	141.9	142
Serum Cl (99–110 mmol/L)	111↑	107	107.2	108.7
Serum Ca (2.11–2.52 mmol/L)	2.07↓	2.27	2.32	2.36
Serum Mg (0.75–1.02 mmol/L)	0.77	0.79	0.85	0.81
Serum P (0.85–1.51 mmol/L)	0.29↓	0.61↓	0.69↓	1.18
Urinary P (12–42 mmol/24 h)	15.75	–	–	–
Urinary Ca (2.5–7.5 mmol/24 h)	1.98↓	–	–	–
Glycosuria	Negative	Negative	Negative	–
Proteinuria	Negative	Negative	Negative	–
TRP (85–95%)	58.07%↓	–	–	–
TmP/GFR (0.80–1.35 mmoL/L)	0.17↓	–	–	–
ALP (30–120 U/L)	214↑	194↑	209↑	272↑
PTH (15–65 pg./mL)	150↑	83↑	53	47.5
25(OH)D (20–50 ng/L)	18.9↓	15.4↓	–	20.1

K, potassium; Na, sodium; Cl, chlorine; Ca, calcium; Mg, magnesium; P, phosphorus; TRP, tubular reabsorption of phosphate; TmP/GFR, tubular maximum reabsorption of phosphate to glomerular filtration rate; ALP, alkaline phosphatase; PTH, parathyroid hormone; 25(OH)D, 25 hydroxy vitamin D; ↑, above normal levels; ↓, below normal levels.

As TIO was suspected, a whole-body ^68^Ga-DOTA-TATE PET/CT was performed, revealing a focal area of abnormally increased ^68^Ga-DOTA-TATE uptake in the left temporal region, raising concerns about a neoplastic lesion (Figures 1C–F). Enhanced magnetic resonance imaging (MRI) demonstrated a well-circumscribed, T2-hyperintense, and homogeneously enhancing nodule (7 mm * 10 mm) within the left temporalis muscle, with no associated bone erosion (Figures 1G,H). However, the patient had no temporal symptoms.

The tumor was radically excised through surgery. During the procedure, the primary surgeon created a 4-cm incision in the patient’s left temporal region. After layered dissection, a firm mass measuring 1 cm × 1 cm, which was adherent to the temporalis muscle, was identified and completely excised using electrocautery. The surface of the temporal bone was rough with spiculated projections, which were smoothed out with a burr. The subsequent histopathological analysis confirmed the tumor as a PMT, strongly supported by diffuse immunoreactivity positive for special AT-rich sequence-binding protein 2 (SATB2), neural cell adhesion molecule (CD56), and somatostatin receptor type 2 (SSTR2), while other markers tested negative includes ETS-related gene (ERG), pan-cytokeratin (CK-PAN), Desmin, S100 protein, progester (PR), and alpha-smooth muscle actin (α-SMA) (Figure 2). Postoperatively, the patient exhibited further improvement in joint pain and range of motion, alongside a gradual normalization of his serum phosphorus level, which reached 1.18 mmol/L by day 27. The patient showed no evidence of recurrence at the 6-month follow-up.

**Figure 2:**
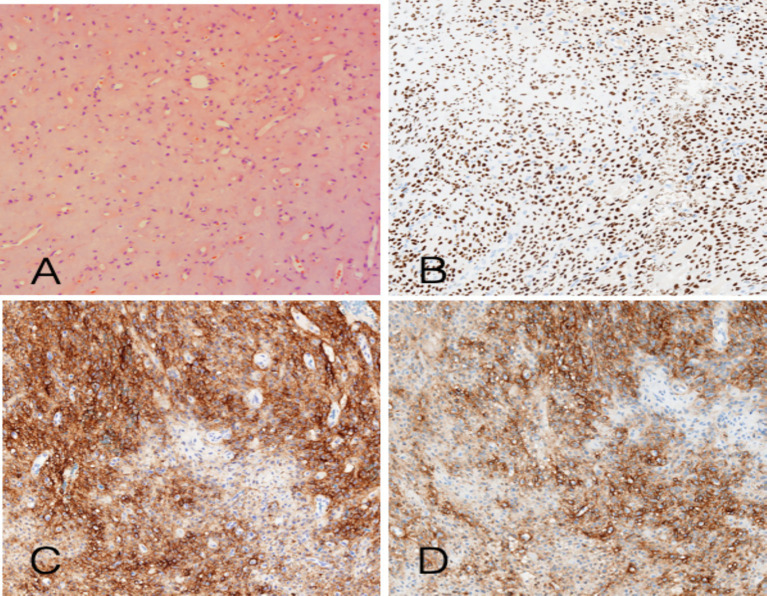
The pathology and immunohistochemistry of this case. The tumor is rich in vessels **(A)**. Expression of SATB2 **(B)**, CD56 **(C)**, and SSTR2 **(D)** (x40).

## Discussion

This case presents a 54-year-old man with TIO caused by a PMT in the left temporalis muscle, manifesting as long-term polyarthralgia, impaired bone healing, osteonecrosis in the femoral head, and multiple pathological fractures.

The PMTs are often small, slow-growing tumors that can be found anywhere in the body—from head to toe (3, 10). The distribution analysis in a study found that the lower extremities are the predominant sites (52.9%), followed by the head and neck region (30.0%) and the chest (6.7%) (7). Among these sites, the hip was the most susceptible site for tumor formation (25.6%) (11), and the femur is also a common site (12). Within the head and neck region, the sinonasal region is the common site, followed by the mandible and maxilla (13). These sites should be carefully examined explored when the tumor is not easily detectable. Patients with PMT are often associated with a history of osteomalacia, as reported in this case. In the early stages, patients may present with typical manifestations of osteomalacia, including bone pain, muscle weakness, and multiple fractures (5). TIO caused by PMTs often presents as non-specific musculoskeletal symptoms, which are easily misdiagnosed as osteoporosis, rheumatic diseases, or neuromuscular lesions (14). The misdiagnosis rate is up to 95.1% (10). In the present case, the tumor was diagnosed and precisely localized after a 2-year delay.

The typical pathophysiological characteristic is the overproduction of FGF-23, a hormone primarily secreted by osteoblasts and osteocytes (15). This hormone disrupts calcium and phosphate homeostasis through a dual mechanism (16). First, this hormone directly downregulates the production of type 2a/2c sodium–phosphate cotransporters in the proximal tubules and inhibits phosphate reabsorption. Second, FGF23 reduces intestinal phosphate absorption by modulating vitamin D metabolism. The majority of TIO cases are associated with increased FGF23 levels (6, 8), although some patients present with normal FGF23 levels (7). Although FGF-23 was not measured in this case, PMT should still be highly suspected. The serum phosphate level in this case was significantly decreased, while ALP and PTH levels were elevated. FGF-23 induces renal phosphate wasting and impairs calcitriol synthesis, leading to poor bone mineralization and multiple pathological fractures, as observed in this patient. Additionally, increased bone turnover results in elevated ALP levels. Furthermore, the complete resolution of hypophosphatemia and improvement in symptoms following tumor resection provide strong indirect evidence of FGF-23 involvement.

Fanconi syndrome is another cause of renal phosphate wasting. However, in this case, the patient had no glycosuria, no clinical manifestations of proximal renal tubular acidosis, and no history of nephrotoxic drug exposure. Serum potassium levels remained normal throughout the treatment course. Although aminoaciduria and bicarbonate (HCO_3_^−^) data were not available, the absence of these results did not affect the final diagnosis of TIO. Given the patient’s low levels of 25(OH)D, mild hypocalcemia, and elevated PTH, vitamin D deficiency along with secondary hyperparathyroidism should also be considered. However, the patient also exhibited severe renal phosphate wasting (TRP 58.07%, TmP/GFR 0.17 mmol/L), which cannot be explained by vitamin D deficiency alone. Meanwhile, the serum phosphorus level was only partially improved with medical therapy, but complete normalization was possible only after tumor resection, and the PTH levels also normalized rapidly following tumor removal.

Locating the responsible tumors using the conventional anatomical imaging modalities (such as CT or MRI) is quite challenging. To enhance the diagnostic accuracy for TIO, functional imaging techniques are widely used. In the reported study, the detection rate was highest for ^68^Ga-DOTA-based PET/CT (98.9%), followed by a technetium-99 m hydrazinonicotinamide-octreotide (Tc99m-HYNIC-octreotide) scan (95.3%) (3). These findings highlighted the effectiveness and potential of PET/CT imaging in suspected cases. In this case, the lesion within the temporalis muscle was detected through ^68^Ga-DOTA-TATE PET/CT. Subsequent enhanced MRI further revealed the anatomical location and size of the lesion. Thus, a stepwise imaging strategy—starting with functional imaging followed by anatomical imaging—is recommended for tumor localization.

However, when using the above functional imaging techniques, it is important to note that multiple areas of tracer avidity may be observed. These areas may represent true tumor sites, but false-positive uptake may also occur due to inflammation or bone fractures (17, 18). In these ambiguous cases, selective venous sampling for FGF-23 can help localize the culprit tumor, with a sensitivity ranging from 83 to 94% (19, 20). This technique involves collecting blood samples from multiple sites across the body to measure FGF-23 levels. Theoretically, the tumor is located near the site where the FGF-23 concentration is highest (21, 22).

Curative surgical resection with wide margins is the established standard of care and the only definitive therapy available (11). Once the tumor responsible for the condition is removed, phosphate concentrations gradually increases and returns to the normal level within an average of 5 days, and symptoms begin to alleviate within a few days or weeks, but complete relief may take several months (23). In this case, serum phosphate and PTH levels normalized within 27 days, except for ALP levels. The elevated ALP levels may be related to the initiation of skeletal repair, characterized by a marked proliferation and enhanced activity of osteoblasts. As a functional marker of osteoblasts, ALP may be increased due to the hypermetabolism of bone matrix. Coman et al. (11) reported thst ALP in 76 TIO patients returned to normal levels approximately 18 months after surgery. In addition, significant improvement in BMD may be achieved within 2–4 years after surgery (4, 24).

The PMT is classified into four histological subtypes, namely mixed connective tissue, osteoblast-like, non-ossifying fibroma-like, and ossifying fibroma-like, in which mixed connective tissue subtype accounts for almost 90%. In this study, the pathological findings showed submucosal small vessel proliferation and spindle cells. Immunohistochemistry demonstrated positivity for SATB2, CD56, and SSTR2. Combined with the clinical presentation of hypophosphatemia and osteomalacia, a diagnosis of PMT was supported.

In cases where complete tumor resection or detection is not feasible, drug treatment remains critical. Conventional drug treatment includes calcium, phosphate, and calcitriol supplementation, with the aim of correcting phosphate and vitamin D imbalance, alleviating symptoms, and normalizing bone mineralization. The complete normalization of serum phosphate levels may increase the risk of secondary and hyperparathyroidism; therefore, it is recommended to maintain the serum phosphate levels at the lower limit of the normal range (25). Burosumab, a fully human monoclonal antibody against FGF-23, has been approved for treating TIO in cases of inoperable, recurrent, or non-localizable tumors (26, 27). In Phase II clinical trials, this drug has shown promising clinical application prospects with a favorable safety profile (26).

This case has several limitations. First, the serum FGF-23 levels were not measured. At the initial diagnosis, the clinicians did not suspect TIO, as the patient’s clinical manifestations were attributed to other diseases, including arthritis, osteoporosis, and osteonecrosis of the femoral head. Second, the 24-h postoperative urine phosphorus, TRP, and TmP/GFR data were unavailable because the patient’s serum phosphorus levels normalized rapidly after surgery and simultaneous urine samples were not collected. Future studies should strengthen the process of monitoring these indicators, including serum FGF-23 and renal phosphorus metabolism parameters, in order to improve the accuracy of disease assessment.

## Conclusion

The subtle presentation of temporal PMT often leads to missed diagnosis, resulting in delays and reduced quality of life. Consequently, a high level of clinical vigilance is required. PMTs should be considered in patients with persistent hypophosphatemia, unexplained bone pain, muscle weakness, or fractures to prevent diagnostic delays and improve outcomes.

## Data Availability

The original contributions presented in the study are included in the article/supplementary material, further inquiries can be directed to the corresponding authors.
